# Electrophysical Properties of the Three-Component Multiferroic Ceramic Composites

**DOI:** 10.3390/ma17010049

**Published:** 2023-12-22

**Authors:** Dariusz Bochenek, Przemysław Niemiec, Dagmara Brzezińska, Grzegorz Dercz, Marcin Wąs

**Affiliations:** 1 Institute of Materials Engineering, Faculty of Science and Technology, University of Silesia in Katowice, 75 Pułku Piechoty 1a, 41-500 Chorzów, Poland; dariusz.bochenek@us.edu.pl (D.B.); grzegorz.dercz@us.edu.pl (G.D.); 2 Department of Bioprocess Engineering, Power Engineering and Automations, University of Agriculture in Krakow, Balicka 120, 31-120 Krakow, Poland; marcin.was@urk.edu.pl

**Keywords:** multiferroic composites, multiferroics, ferroelectrics, phase transition

## Abstract

Using the free (pressureless) sintering method, multiferroic ceramic composites based on two ferroelectric materials, i.e., BaTiO_3_ (B) and Pb_0.94_Sr_0.06_ (Zr_0.46_Ti_0.54_)_0.99_Cr_0.01_O_3_ (P), and magnetic material, i.e., zinc–nickel ferrite (F) were obtained. Three composite compositions (BP-F) were obtained with a constant 90/10 content (ferroelectric/magnetic) and a variable content of the ferroelectric component (B/P), i.e., 70/30, 50/50, and 30/70. Crystalline structure, microstructural, DC electrical conductivity, dielectric, and ferroelectric properties of multiferroic composites were investigated. The concept of a composite consisting of two ferroelectric components ensures the preservation of sufficiently high ferroelectric properties of multiferroic composites sintered by the free sintering method. Research has shown that the percentage of individual ferroelectric components in the composite significantly affects the functional properties and the entire set of physical parameters of the multiferroic BP-F composite. In the case of the dielectric parameters, the best results were obtained for the composition with a more significant amount of BaTiO_3_; i.e., permittivity is 1265, spontaneous polarization is 7.90 µC/cm^2^, and remnant polarization is 5.40 µC/cm^2^. However, the most advantageous set of performance parameters shows the composite composition of 50BP-F.

## 1. Introduction

In recent years, well-known ceramic materials with functional properties, e.g., [1,2,3,4,5,6], have constituted various engineering materials with versatile applications in modern microelectronics [7,8]. These materials include multiferroic composite materials obtained based on ferroelectric and magnetic compounds [8,9,10,11,12,13]. Multiferroics are commonly used in modern applications, e.g., actuators, amplifiers, sensors, micro-shifters, memory elements, magnetoelectric transducers, piezoelectric transducers, energy storage devices, and harvesting [11,14,15,16,17,18,19,20].

The functional properties of ceramic materials strongly depend on the quality of the synthesized powders obtained as a result of their synthesis, as well as on the perfection of grain crystallization in the ceramic microstructure obtained during sintering in the technological process. Chemical homogeneity, degree of crystallization, and grain size (average grain size, uniformity) are significant [1,21,22]. The sintering process should ensure, among other things, the completion of the synthesis processes, proper crystallization of the grains, their strong bonding and the gradual disappearance of porosity, the disappearance or reduction in internal stresses in the grains, and the formation of the structure of boundaries of adjacent grains. Most of the above factors are related to diffusion processes, and their activation is caused by both external factors (i.e., temperature, pressure) and internal factors (i.e., admixtures, deviations from compositional stoichiometry, various types of defects, e.g., vacancies) [21,22]. The sintering process of ferroelectric materials occurs at high temperatures, i.e., in the paraelectric phase. When it is cooled to room temperature, a phase transformation occurs, which involves spontaneous deformation of the unit cell and the formation of a more or less complex domain structure. Therefore, in addition to the sintering temperature and time, the sinter cooling rate also becomes an essential parameter of the sintering process. In ceramic materials, the ferroelectric state is very susceptible to structural defects, including cation and oxygen vacancies. In compounds containing lead, these are most often lead vacancies, while in all oxide ferroelectrics, long-term sintering at high temperatures causes the appearance of oxygen vacancies. One way to reduce excessive defect formation in the ceramic material is to reduce the sintering temperature and time [21,22].

Many sintering methods are known to obtain optimal properties of ferroelectric and multiferroic materials, e.g., microwave sintering, spark plasma sintering, hot pressing, pressureless free sintering, and cold sintering process [23,24,25,26,27]. For each sintering method used, appropriate optimization of the process conditions is made, but each has advantages and disadvantages. The free sintering method is the most common and economical method of compacting multiferroic composite powders [26,28,29]. In designing multiferroic ceramic composites (as a ferroelectric component), materials with high ferroelectric and piezoelectric properties, such as PZT-type solid solution [5] and BaTiO_3_ [30], were considered.

The most famous solid solution with a wide range of functional properties and applications in microelectronics is Pb (Zr_1−*x*_Ti*_x_*)O_3_ (PZT), created from the combination of PbTiO_3_ and PbZrO_3_ [31]. The PbTiO_3_ compound is one of the model ferroelectrics that has the symmetry of a tetragonal system at room temperature, while the PbZrO_3_ compound is an antiferroelectric with an orthorhombic deformation of the regular unit cell. The PZT solid solution has a perovskite structure (with the general formula ABO_3_), where titanium Ti^4+^ and zirconium Zr^4+^ cations alternately occupy the B positions in the unit cell, whereas lead Pb^2+^ cations occupy the A positions. In turn, oxygen O^2−^ anions form so-called oxygen octahedra surrounding the cations from position B. The physical properties (i.e., dielectric, piezoelectric, pyroelectric) of the PZT solid solution and the possibilities of its practical applications depend on many factors. Among them are the Zr/Ti concentration ratio in the base composition, the type and concentration of dopants introduced into both the A and B positions of the unit cell, as well as the crystal structure and microstructure, which are determined by the technological process conditions used [31,32]. In the Zr/Ti percentage range from 100/0 to 96/4, the PZT has rhombic symmetry and functions as an antiferroelectric material (i.e., the material does not exhibit the piezoelectric effect). In the Zr/Ti range from 96/4 to 53/47, PZT is a ferroelectric material with a rhombohedral structure. In contrast, in the range from 53/47 to 0/100, it is a ferroelectric material with a tetragonal structure. The phase transition temperature from the ferroelectric to the paraelectric phase increases with the increase in the amount of titanium in the PZT composition. Exciting functional properties of PZT-type ceramic materials (with extremely high or extremely low parameters) occur in the morphotropic area, i.e., in the area of a mixed tetragonal and rhombohedral phase [31,33,34].

A characteristic feature of PZT is also its wide isomorphism, which allows for the substitution of appropriate cations in the A and B positions of the compound [21,35,36,37]. There are several doping methods, e.g., soft (donor) doping, hard (acceptor) doping, soft–hard doping, and middle–hard doping [1,21,38]. The cations of the soft ferroelectric dopant have a higher valence than the PZT base cations, for example lanthanum La^3+^, neodymium Nd^3+^, antimony Sb^3+^, and bismuth Bi^3+^ (in the A positions of the unit cell) and niobium Nb^5+^, tantalum Ta^5+^, antimony Sb^5+^, and tungsten W^6+^ (in the B positions). The consequence of soft PZT doping is the formation of cation vacancies in the crystal lattice, increasing the permittivity (*ε*), electrical resistivity (*ρ*_v_), electromechanical coupling coefficient (*k*_p_), components of the elastic compliance tensor (*S*_ij_), and internal friction, whereas reducing the coercivity field (*E*_c_), mechanical quality (*Q*_m_), and electrical quality (*Q*_e_). In contrast, hard dopant cations have a lower valence than the substituted PZT base cations; therefore, oxygen vacancies are created in the crystal lattice to maintain electrical neutrality [37]. These most often include potassium K^+^, sodium Na^+^ (in the A positions of the unit cell), iron Fe^3+^, chromium Cr^3+^, aluminum Al^3+^, scandium Sc^3+^, and indium In^3+^ (in the B positions). Hard admixtures increase the ferroelectric hardness of PZT piezoceramics, i.e., reduce the values of the following parameters *ε*, dielectric loss tangent tan*δ*, *k*_p_, and *ρ*_v_. In contrast, *E*_c_, *Q*_m_, and *Q*_e_ parameters increase. The multi-component solid solutions based on PZT resulting from doping are characterized by ferroelectric, dielectric, and piezoelectric parameters that are much better than the basic composition [21]. As the number of components of the PZT solid solution increases, the morphotropic area is expanded, raising the possibility of selecting ceramic materials with the desired dielectric and piezoelectric parameters [21,33,35].

The chemical composition of Pb (Zr_1−*x*_Ti*_x_*)O_3_ materials is never perfectly stoichiometric, as it usually contains oxygen vacancies and (or) lead vacancies formed due to the sintering process. The electrical conductivity type, i.e., the type of majority free charge carriers of perovskite materials, depends on the concentration ratio of cationic and anionic vacancies. If anionic vacancies predominate over cationic vacancies in the crystal lattice, the material exhibits electronic conductivity (n-type), while if cationic vacancies predominate, the material exhibits hole conductivity (p-type) [31]. Undoped PZT ceramics exhibit p-type electrical conductivity, which is the result of its increased oxidation, creating an excess of lead (cationic) vacancies over oxygen (anionic) vacancies [1,21]. The resulting vacancies in the A positions attract electrons to ensure the reconstruction of the electron shells surrounding oxygen ions. Thus, by changing the Zr/Ti ratio in PZT, it is possible to obtain piezoceramic materials for specific applications in many different fields of microelectronics but with entirely different purposes (e.g., broadband electrical filters, piezoelectric transformers, sensors, micro-sliders, micro-actuators, vibrators, ultrasound generators), e.g., [1,22].

Ferroelectric material BaTiO_3_ (BT), which is characterized by a very high electrical resistance (>10^8^ Ωm), is also among the group of ferroelectric materials that have been most studied in recent decades [30,39]. In a typical perovskite structure, barium Ba^2+^ ions occupy the corner positions of the unit cell (site A), and titanium Ti^4+^ ions are located in the center of the unit cell (site B). In contrast, oxygen ions O^2−^ occupy wall-centered positions on the cube’s walls, which create the oxygen octahedra. The acute ferroelectric phase transition of BT ceramics from the ferroelectric to the paraelectric phase occurs at a temperature close to 120 °C [30]. A characteristic feature of BaTiO_3_ ceramics is that at different temperatures, its crystal structure undergoes changes and structural distortions, as a result of which the BT material can occur in cubic, hexagonal, trigonal, orthorhombic, and tetragonal phases [30]. BaTiO_3_ belongs to the family of semiconductor ceramic materials with active grain boundaries, i.e., with a sharp increase in the specific resistance of ceramics above the ferroelectric Curie point (Positive Temperature Coefficient of Resistance, PTC-R). The PTC-R effect occurs in ferroelectric semiconductor materials and depends on their chemical composition and production conditions, which determine the activity of intergrain boundaries. Similarly to PZT, appropriate doping of the basic composition of the BT material makes it possible to improve its functional properties and expand its potential applications, e.g., in energy storage systems and devices. For example, work [39] examined the influence of bismuth Bi^3+^, magnesium Mg^2+^, zinc Zn^2+^, tantalum Ta^5+^, and niobium Nb^5+^ admixtures introduced into the BT-like solid solution to improve the breakdown strength and energy storage efficiency and reduce the Curie temperature.

During designing multiferroic composite materials, various types of ferrite materials with different properties are often selected as magnetic components, e.g., nickel, cobalt, cobalt–zinc, zinc–manganese, nickel–zinc, and cobalt–zinc–manganese [9,40,41,42]. An undesirable effect of introducing ferrite is most often a deterioration of ferroelectric properties and an increase in the electrical conductivity of multiferroic ceramic composites [10]. Therefore, the appropriate selection of the magnetic component and its amount in the multiferroic composite composition is mainly based on the high resistance of ferrite at room temperature and, at the same time, having high magnetic parameters.

In this work, the results of three compositions of multiferroic composite materials obtained based on magnetic zinc–nickel ferrite and two ferroelectric materials BaTiO_3_ (B) and Pb_0.94_Sr_0.06_ (Zr_0.46_Ti_0.54_)_0.99_Cr_0.01_O_3_ (P) were obtained and presented. The ferroelectric/ferrite percentage was 90/10 (constant for the whole series of samples), while the ferroelectric component (B/P) content was variable, i.e., 70/30, 50/50, and 30/70. Studies on the effect of changing the composition of the ferroelectric composite matrix on the electrophysical properties of multiferroic composite materials were carried out.

## 2. Experiment

### 2.1. Material and Technology Process

The multiferroic composites under study consisted of two main components, i.e., magnetic (Ni_0.64_Zn_0.36_Fe_2_O_4_ ferrite material) and ferroelectric materials. For the ferroelectric component, two materials were used simultaneously, i.e., BaTiO_3_ (B) and Pb_0.94_Sr_0.06_ (Zr_0.46_Ti_0.54_)_0.99_Cr_0.01_O_3_ (P) with different percentages, namely, 70/30, 50/50, and 30/70. The composites were obtained in proportion of 90% of the ferroelectric phase (B+P) to 10% of the magnetic (F) phase.

The Pb_0.94_Sr_0.06_ (Zr_0.46_Ti_0.54_)_0.99_Cr_0.01_O_3_ (P) powder was obtained using solid-state reaction method using PbO (99.99%, POCH, Gliwice, Poland), ZrO_2_ (99.5%, Sigma-Aldrich, St. Louis, MO, USA), TiO_2_ (99.99%, Sigma-Aldrich, St. Louis, MO, USA), Cr_2_O_3_ (99.99%, POCH, Gliwice, Poland), and SrCO_3_ (99.99%, Chempur, Piekary Śląskie, Poland) reagents. The technology process of the P material was carried out according to the reaction equation: 0.94 PbO + 0.06 SrCO_3_ + 0.4554 ZrO_2_ + 0.5346 TiO_2_ + 0.005 Cr_2_O_3_ → Pb_0.94_Sr_0.06_ (Zr_0.46_Ti_0.54_)_0.99_Cr_0.01_O_3_ + 0.06 CO_2_↑. The PbO was weighed in excess (5%). The input powders were mixed using a planetary ball mill (Fritsch Pulverisette 6, Idar-Oberstein, Germany) via a wet method in ethyl alcohol for 12 h, while synthesis was performed under 850 °C/2 h conditions. BaTiO_3_ (B) commercial material (99.5%, Sigma-Aldrich, St. Louis, MO, USA) has been used. The Ni_0.64_Zn_0.36_Fe_2_O_4_ ferrite (F) was obtained using NiO (99.99%, POCH, Gliwice, Poland), Fe_2_O_3_ (99.9%, POCH, Gliwice, Poland), and ZnO (99.99%, POCH, Gliwice, Poland) reagents. The ferrite material was obtained according to the following reaction equation: 0.64 NiO + 0.36 ZnO + Fe_2_O_3_ → Ni_0.64_Zn_0.36_Fe_2_O_4_. The input powders were mixed using a planetary ball mill (Fritsch Pulverisette 6, Idar-Oberstein, Germany) via a wet method in ethanol for 24 h, and the next mixture of powders was calcined at 1000 °C/4 h.

Three compositions of composite materials BP-F were obtained, in which the ferroelectric component of the composite material (B and P) had different proportions, i.e., 70% B/30% P, 50% B/50% P, and 30% B/70% P. On the other hand, a constant 90/10 proportion of composite materials’ ferroelectric (B+P) and magnetic (F) components were selected. For each multiferroic composite composition, the ferroelectric and magnetic powders were weighed appropriately and mixed in a planetary ball mill (Fritsch Pulverisette 6, Idar-Oberstein, Germany) via a wet method for 24 h. The mixed powders were calcined at 1050 °C for 4 h. Then, the composite powders were pressed on an MP250M semi-automatic press (ATEST, Kielce, Poland) at a pressure of 300 MPa, obtaining disc-shaped moldings with a diameter of 10 mm. The sintering of ceramic samples was carried out under conditions of 1250 °C/4 h via the free sintering method. After sintering, the sample surfaces were ground and polished to a thickness of 1 mm and then annealed to remove mechanical stresses. The last stage of the technological process was to apply silver paste to both surfaces of the samples in order to conduct electrical tests. The BP-F multiferroic composite samples were marked as 70BP-F, 50BP-F, and 30BP-F, respectively.

### 2.2. Characterization

A Philips X’Pert PW 3040/60 diffractometer (copper radiations CuK*_α_*_1_/*_α_*_2_) was used for the X-ray analysis of the multiferroic composite powders. The measurements were conducted at room temperature (RT) in the range of 2*θ* angles from 15° to 100°, whereas phase identification was performed according to the ICDD PDF-4 database. Energy dispersive spectrometry (EDS), electron probe microanalysis (EPMA), and microstructure (SEM) of the multiferroic composites were performed using a scanning electron microscopy JSM-7100F TTL LV (Jeol Ltd., Tokyo, Japan). Spot and surface EDS analyses were performed to determine the multiferroic composites’ basic composition.

Temperature dielectric studies were conducted on the LCR Meter (QuadTech 1920 Precision, Maynard, MA, USA). The permittivity and dielectric loss factor tests were performed for frequencies ranging from 20 Hz to 1 MHz (for 45 frequencies) at a heating rate of 2°/min, in the temperature range from room temperature to 500 °C. DC conductivity was carried out on the electrometer (Keithley 6517B, Cleveland, OH, USA). *P-E* hysteresis loops were made using a Sawyer–Tower circuit with a voltage amplifier (Matsusada Inc. HEOPS−5B6 Precision, Kusatsu, Japan) at room temperature RT (5 Hz, 3.5 kV/mm). Dielectric parameter measurements were recorded in a control program written in the LabView environment, and a NI USB-6002 digital transducer card (National Instruments Corporation, Austin, TX, USA) captured the measurement data.

## 3. Results

**Figure 1:**
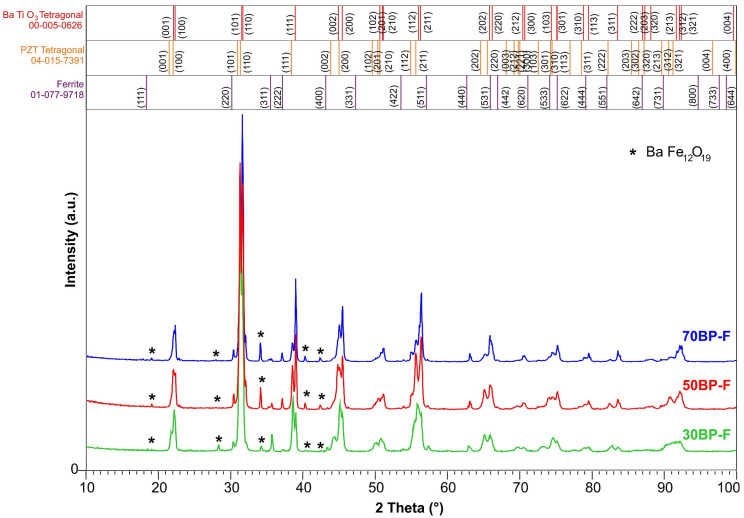
X-ray diffraction patterns of the BP-F composite materials and their individual components, i.e., BaTiO_3_ (B), Pb_0.94_Sr_0.06_ (Zr_0.46_Ti_0.54_)_0.99_Cr_0.01_O_3_ (P), and Ni_0.64_Zn_0.36_Fe_2_O_4_ (F).

X-ray crystal structure studies of multiferroic ceramic composites after the sintering process are presented in Figure 1. X-ray measurements showed the set of reflections coming from three main components of the multiferroic composites, i.e., two from ferroelectric phases BaTiO_3_ and PZT and a third from the magnetic ferrite phase (Ni_0.64_Zn_0.36_Fe_2_O_4_). The multiferroic composite samples were identified based on the ICDD PDF-4 database (International Center for Diffraction Data Powder Diffraction Files). The best match between patterns and experimental results was obtained for card no. ICDD 00-005-0626 (tetragonal phase with a P4*mm* space group) for BaTiO_3_ (B), card no. ICDD 04-015-7391 (tetragonal phase and P4*mm* space group) for PZT-type material (P), and card no. ICDD 01-077-9718 (cubic crystal system and F*d*$\bar{3}$*m* space group) for ferrite material Ni_0.64_Zn_0.36_Fe_2_O_4_. The X-ray studies also revealed the presence of an additional phase, i.e., BaFe_12_O_19_ (card no. ICDD 04-002-2503). Figures S1–S3 (in the Supplementary Materials) present the results of X-ray examinations of the individual composite components, i.e., BaTiO_3_ (B), Pb_0.94_Sr_0.06_ (Zr_0.46_Ti_0.54_)_0.99_Cr_0.01_O_3_ (P), and Ni_0.64_Zn_0.36_Fe_2_O_4_ (F), before combining them to form a composite material. In the case of ferroelectric components B and P, single-phase materials were obtained (without foreign phases). However, X-ray analysis of the ferrite material (F) revealed the presence of trace amounts of unreacted Fe iron in addition to the main phase. The excess iron (under favorable sintering conditions) quite easily combines with barium (Ba) to form the complex compound BaFe_12_O_19_, creating an additional phase in composite materials.

Structure refinement using the Rietveld method based on XRD results was performed for three BP-F composite samples (Table 1). The tests showed changes in the lattice parameters of individual composite phases when their percentage content in the composite chemical composition changed. In the 70BP-F composition, the unit cell parameters have higher values than the 30BP-F composition. Moreover, in analyzing individual phases of the composite material for BaTiO_3_, the unit cell expanded compared to the standard parameters (card no. ICDD 00-005-0626). In the case of the tetragonal PZT-type phase, the determined lattice parameters indicate the contraction of the unit cell (*a*_0_ and *b*_0_ are smaller, and *c*_0_ are higher compared to the standard parameters (card no. ICDD 04-015-7391)). In turn, for the ferrite material Ni_0.64_Zn_0.36_Fe_2_O_4_, deviations of the *a*_0_ parameter from the standard value (card no. ICDD 01-077-9718) are observed, indicating the unit cell’s contraction. A similar tendency was observed for the foreign phase BaFe_12_O_19_, where the lattice parameters *a*_0_ and *b*_0_ were lower than the catalog data. At the same time, the *c*_0_ parameter was higher (the BaFe_12_O_19_ unit cell also showed contraction).

**Figure 2:**
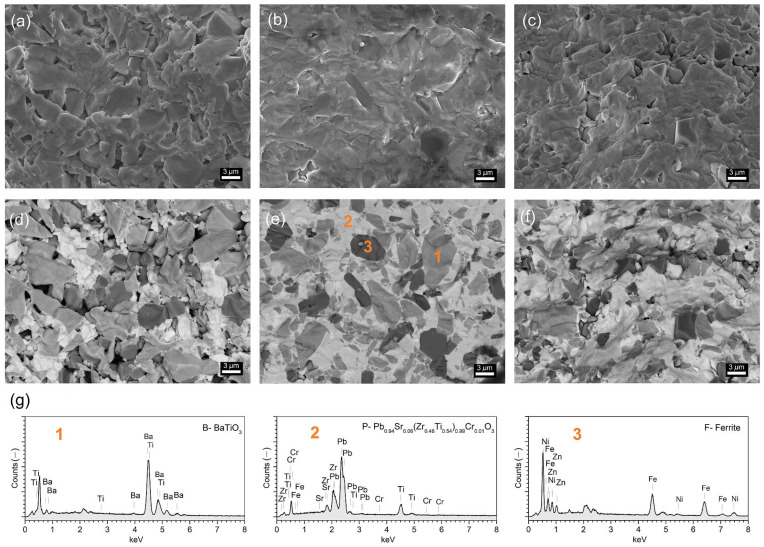
SEM microstructure of the BP-F multiferroic composites: 70BP-F (**a**,**d**), 50BP-F (**b**,**e**), 30BP-F (**c**,**f**); figures (**a**–**c**) image captured by the SB technique; figures (**d**–**f**) image captured in the BSE technique; graphs below (**g**), the spot EDS analysis of the BaTiO_3_ (1), Pb_0.94_Sr_0.06_ (Zr_0.46_Ti _0.54_)_0.99_Cr_0.01_O_3_) (2), and ferrite (3).

Figure 2 presents images of the cross-section of the surface microstructure for ceramic composite materials. The SEM images were performed using the SB technique (detection of the signals from the secondary and backscattered electron detectors) and the BSE technique (detection of backscattered electrons). The BSE technique made it possible to separate the areas originating from the magnetic phase (dark areas) and the ferroelectric phase, with the separation of areas with BaTO_3_ grains (grey areas) and PZT ones (light areas). The affiliation of the individual phases was confirmed by the spot EDS analysis (Figure 2g) with the appropriate designation of the analysis 1–B, 2–P, 3–F). The highest homogeneity of the microstructure and low porosity of the composite samples were obtained for the composition with the exact content of ferroelectric components B/P, i.e., for 50BP-F. The microstructure of the 30BP-F sample shows the highest degree of sintering (solidification) with firmly packed and solidified grains, while the 70BP-F composite sample shows the highest porosity (lowest density)—Table 2.

**Figure 3:**
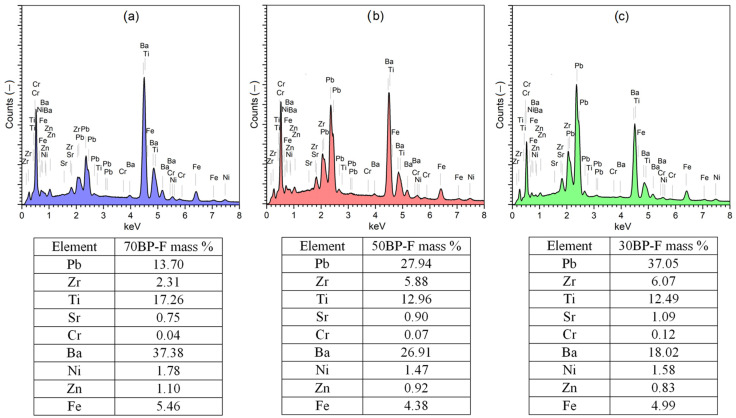
Surface EDS spectrum of multiferroic composites: 70BP-F (**a**), 50BP-F (**b**), and 30BP-F (**c**); tables below present the experimenta percentage of the individual elements of the BP-F composites.

Figure 3 presents surface EDS tests performed on a larger area of ceramic samples. The EDS analysis showed the presence of all component elements in the compositions, an appropriate change in their quantity, and a change in the content of individual composite components (tables in Figure 3). The EDS tests of the multiferroic composite materials confirmed the chemical composition of the multiferroic composite materials close to the designed one. The EDS analysis also confirmed the absence of foreign elements in composite samples. The results of the percentage of individual components of composite samples were the average of five randomly selected areas from the fracture surface of composite samples. The analysis of the distribution of individual elements showed some deviations in the chemical composition between the designed values and their actual content. The 50BP-F composite material has the most significant compliance with the designed composition. The share of the main elements of the individual components of the composite is 99.8% for barium Ba, while for lead Pb and iron Fe, there is an excess (1% and 3.4%, respectively). For the other two composite compositions, the deviation from the designed compositions is higher, namely, for the 70BP-F sample (99.5% for Pb and 94.3% for Ba) and for the 30BP-F sample (98.7% for Pb and 99.7% for Ba) with an excess of Fe. EDS analysis revealed that ferrite is characterized by the lowest stability in the composite compound (in each composite composition, it shows the most significant deviation from the designed composition).

**Figure 4:**
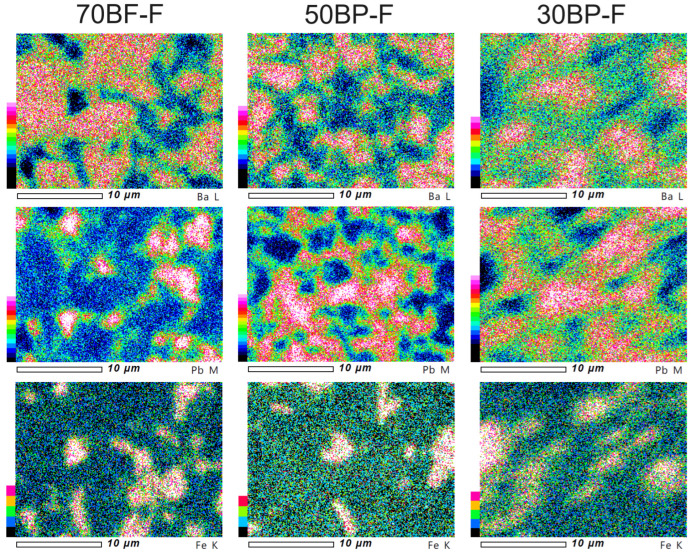
EPMA maps of the BP-F multiferroic composites according to representative elements Ba, Pb, and Fe.

Figure 4 presents the EPMA distribution maps (electron probe microanalysis) for representative elements of the composite samples in the cross-section of the microstructure surface. The EPMA tests took into account the main constituent elements from three composite components, i.e., barium (Ba) for BaTiO_3_, lead (Pb) for Pb_0.94_Sr_0.06_ (Zr_0.46_Ti_0.54_)_0.99_Cr_0.01_O_3_, and iron (Fe) for Ni_0.64_Zn_0.36_Fe_2_O_4_. EPMA mapping revealed the presence of areas originating from individual components of the multiferroic composites, which is consistent with SEM imaging performed using the BSE technique.

**Figure 5:**
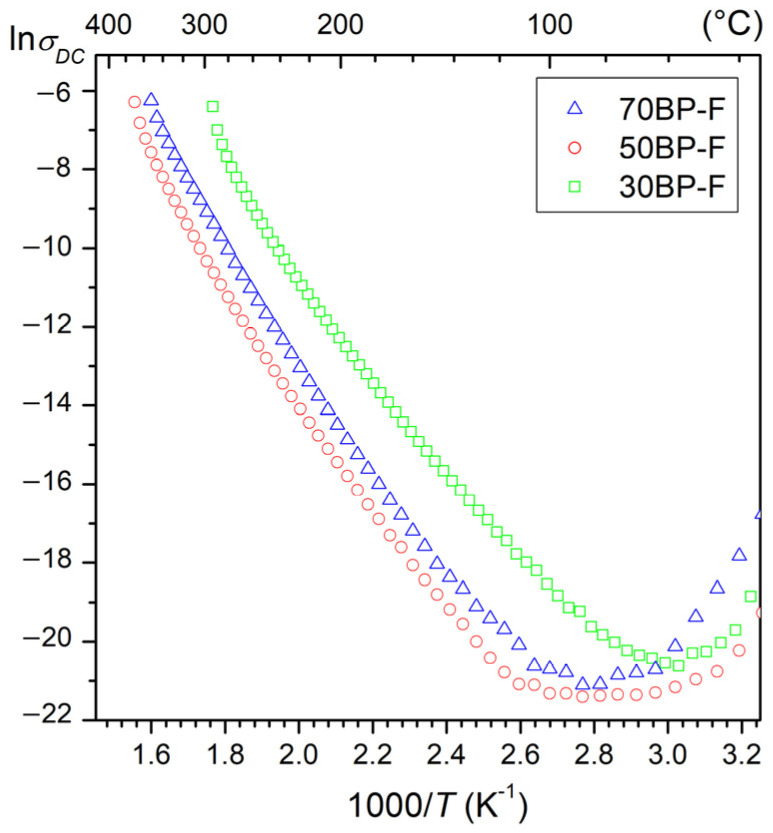
The ln*σ*_DC_ (1000/*T*) graph for BP-F multiferroic composites.

At room temperature, the composite samples have average resistivity values of 5.2 × 10^6^ Ωm for 70BP-F, 1.1 × 10^8^ Ωm for 50BP-F, and 5.4 × 10^7^ Ωm for 30BP-F. Figure 5 shows the temperature dependencies of ln*σ*_DC_ (1000/*T*) for the tested series of composite samples. With increasing temperature, a decrease in the DC electrical conductivity of composite samples is observed. Above 100 °C, there is an increase in electrical conductivity. In the case of composition 30BP-F, this increase occurs at a lower temperature (approx. 80 °C). The calculated activation energies *E*_a_ are in the range from 0.48 to 1.27 eV for the lower temperature range and from 1.00 to 1.17 eV for the higher temperature range. The different activation energy values calculated in the two temperature ranges are related to the different nature of the electrical conductivity depending on various factors. At low temperatures, the ionization processes mainly govern the nature of conductivity, where electrons or holes are the dominating charge carriers. In contrast, at higher temperatures, the mobility of extrinsic defects is responsible for the conductivity [43]. In the case of the multiferroic composites, the calculated *E*_a_ activation energy values indicate that the oxygen vacancies are the dominant factor determining the DC conductivity [44,45].

**Figure 6:**
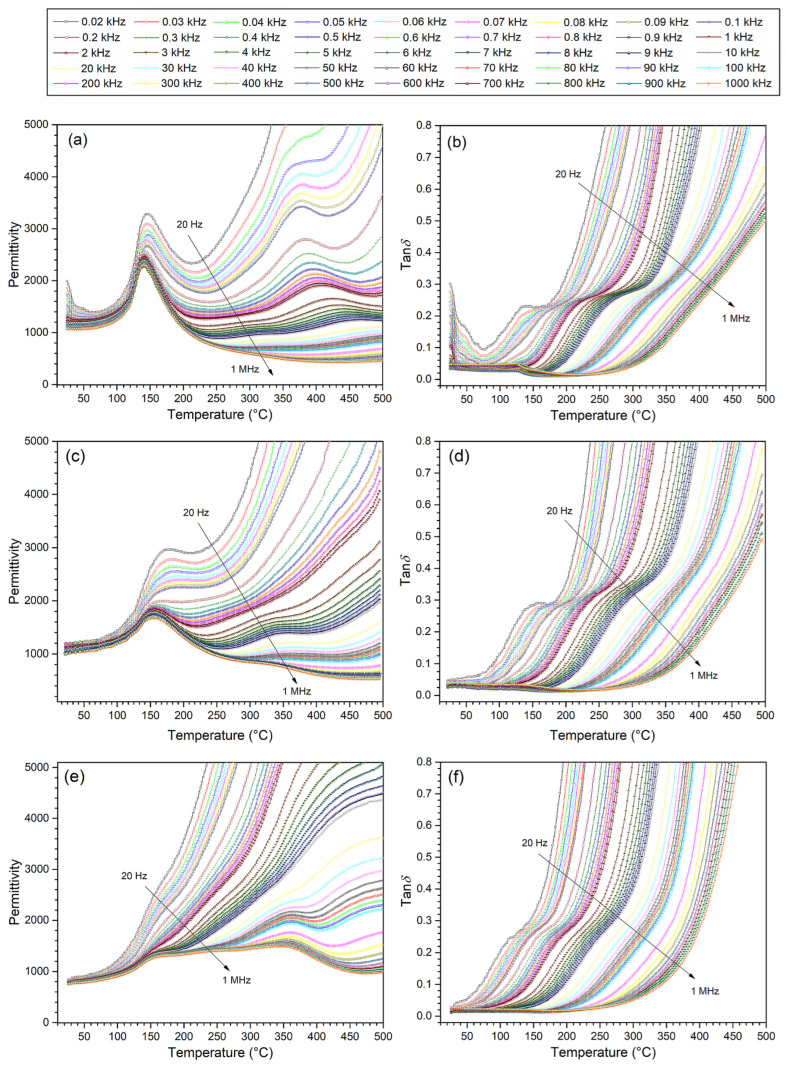
Temperature dependencies of permittivity (**a**,**c**,**e**) and dielectric loss factor (**b**,**d**,**f**) for multiferroic ceramic composites: 70BP-F (**a**,**b**); 50BP-F (**c**,**d**); and 30BP-F (**e**,**f**).

Temperature measurements of permittivity of multiferroic ceramic composites were carried out in the temperature range from room temperature to 500 °C for 45 frequencies and in the frequency range from 20 Hz to 1 MHz (Figure 6). It allowed us to register the occurrence of two local maxima related to the phase transition (ferroelectric–paraelectric) originating from two ferroelectric components of composite materials and their mutual permeation. For some frequency ranges, the characteristic maxima of phase transitions blur and even disappear. Temperature measurements *ε* (*T*) showed a more substantial influence of the BaTiO_3_ material on the charts of dielectric properties. It is due to several factors affecting the temperature permittivity measurement, e.g., at a higher temperature (close to the phase transition of the ferroelectric P), there is also a magnetic phase transition (of the magnetic component—ferrite), which disturbs the dielectric parameters of composite materials, blurring the phase transition of the P component. Clear frequency dispersion is also associated with increased electrical conductivity at high temperatures. In ferroelectric materials, the high-temperature frequency relaxation is related to the space charge of the mechanism conductivity [46,47].

High permittivity values were retained despite the introduction of ferrite material into the composite composition. At room temperature, permittivity values range from 785 to 1265 (Table 2). The acute phase transition maintains the 70BP-F composition for all measurement frequencies used. This is because of the acute phase transition and high permittivity values coming from the BaTO_3_, which is the most abundant in the composite composition. Its strong influence on the dielectric properties is retained and also present in the composition of 50BP-F.

On the other hand, in the case of the 30BP-F composition, for lower frequencies, the blurring of the phase transition is the greatest (the maxima from both phase transitions practically disappear). Local maxima become visible only at frequencies higher than 20 kHz. In the case of this composition, the mutual permeation of two ferroelectric–paraelectric phase transitions is most visible.

**Figure 7:**
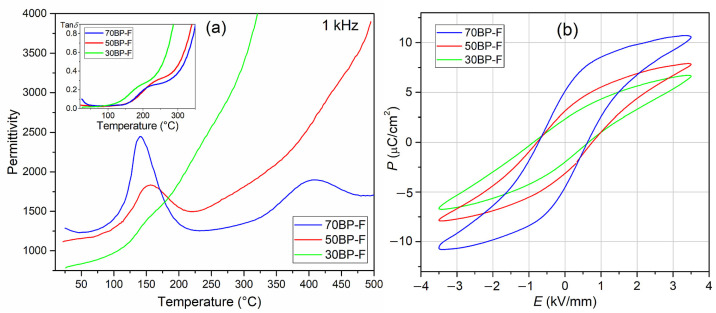
Temperature dependencies of permittivity and tan*δ* (inset) (**a**) and *P*-*E* loops (**b**) measured at room temperature for 5 Hz, and *E* = 3.5 kV/mm for BP-F multiferroic composites.

Figure 7a shows the results of dielectric tests of three composite compositions at 1 kHz. As commonly known, introducing ferrite into composite composition increases the dielectric loss factor values and electrical conductivity [48]. However, in the case of the analyzed compositions, this increase is smaller than in the case of composite materials with cobalt ferrite [28]. At room temperature, the increase in dielectric loss factor (tan*δ*) is most significant for the 70BP-F sample and is 0.1, while for the 50BP-F sample, it is 0.034, and for the 30BP-F composition, it is 0.013. In the case of the 70BP-F sample, an increase in temperature (above room temperature) results in a decrease in dielectric loss factor (to approx. 80 °C), and a further increase in temperature does not cause such a significant increase in tan*δ* values as in the case of the other two 50BP-F and 70BP-F compositions (Figure 6b,d,f and Figure 7a (inside)).

In the case of composite materials, the deterioration of ferroelectric properties is also quite common [48]. Using two ferroelectric materials in a composite composition (one of which has high ferroelectric parameters) may be one of the effective ways to maintain high ferroelectric properties in multiferroic composites with a predominant amount of the ferroelectric phase, also when using classical free sintering [29]. In the case of the analyzed series of BP-F composite samples, hysteresis loops show high saturation (Figure 7b) with an *E*_c_ coercive field in the range from 0.68 to 0.76 kV/mm (for an applied field of 3.5 kV/mm). The highest spontaneous polarization *P*_s_ and remnant polarization *P*_r_ values occur for the 70BP-F composition with the dominant ferroelectric phase BaTiO_3_. They are 5.40 and 7.90 µC/cm^2^, respectively (for an applied field of 3.5 kV/mm). Reducing the percentage of BaTiO_3_ in the ferroelectric composite matrix of the composite material reduces the ferroelectric properties, i.e., *P*_r_ and *P*_s_ values (Table 2, Figure 7b).

## 4. Conclusions

Three compositions of multiferroic ceramic composites composed of ferroelectric (90%) and magnetic (10%) materials were obtained and tested in this work. The composite matrix was a composition of two ferroelectric materials: BaTiO_3_ (B); and Pb_0.94_Sr_0.06_ (Zr_0.46_Ti_0.54_)_0.99_Cr_0.01_O_3_ (P), with variable percentage content (70/30, 50/50, and 30/70). The effect of changing the ferroelectric matrix components on the electrophysical properties of composite materials was investigated. Crystalline structure, microstructural, DC electrical conductivity, dielectric, and ferroelectric properties of multiferroic composites were performed.

The X-ray analysis showed firm maxima derived from ferroelectric components, i.e., B (the tetragonal phase, with a P4*mm* space group) and P (tetragonal phase, with a P4*mm* space group) and the weak ones derived from ferrite material (cubic crystal system, with an F*d*$\bar{3}$*m* space group). The XRD analysis also showed trace amounts of foreign phase (BaFe_12_O_19_), which was formed during the technological process. Studies have shown that using two ferroelectric materials, one of which has high ferroelectric parameters, allows for the maintenance of high ferroelectric properties of composite samples, which are usually significantly worsened by the properties of ferrite. In the case of composition 70BP-F, despite the lower density and higher porosity, the sample shows the highest values of permittivity and remnant polarization. This is due to the most significant amount of the BaTiO_3_ component in the composite material, with an acute phase transition and high permittivity values. These strong properties of the B material are retained in the 50BP-F sample, which has the most advantageous set of all parameters and properties and has the highest density. In turn, the 30BP-F composition shows the highest density and is characterized by the lowest dielectric loss factor values at room temperature.

Multiferroic BP-F ceramic composites built based on magnetic and ferroelectric material show interesting functional properties adequate for applications in modern microelectronics. By controlling the percentage of the ferroelectric component of multiferroic composite materials, it is possible to obtain appropriate final parameters favorable for target applications. In addition, the ferroelectric properties of composite materials can be improved, including by using a PZT-type material from the morphotropic area and doping it with ferroelectric soft dopants.

## Figures and Tables

**Table 1:** The structural analysis performed by the Rietveld method for the BP-F multiferroic composite materials.

Lattice Parameters										
	BaTiO_3_			PZT Tetragonal			BaFe_12_O_19_			Ferrite
	S.G. P4*mm* (99)			S.G. P4*mm* (99)			S.G. P63/*mmc* (194)			S.G. $Fd\bar{3}m$ (227)
	*a_0_* (Å)	*b_0_* (Å)	*c_0_* (Å)	*a_0_* (Å)	*b_0_* (Å)	*c_0_* (Å)	*a_0_* (Å)	*b_0_* (Å)	*c_0_* (Å)	*a_0_* (Å)
ICDD	3.994	3.994	4.0380	4.0260	4.0261	4.1241	5.9000	5.9000	23.300	8.382
70BP-F	4.0406 (4)	4.0406 (4)	4.0614 (6)	3.9911 (3)	3.9911 (3)	4.0216 (8)	5.8925 (4)	5.8925 (4)	23.3982 (3)	8.3529 (1)
50BP-F	4.0394 (4)	4.0394 (4)	4.0602 (6)	3.9915 (2)	3.9915 (2)	4.0213 (4)	5.8875 (6)	5.8875 (6)	23.3673 (5)	8.3533 (1)
30BP-F	4.0193 (2)	4.0193 (2)	4.0922 (3)	3.9930 (2)	3.9930 (2)	4.0293 (4)	5.8732 (5)	5.8732 (5)	24.3085 (4)	8.3528 (1)

**Table 2:** Electrophysical parameters of the BP-F multiferroic composite samples.

Parameter	70BP-F	50BP-F	30BP-F
*ρ* (g/cm^3^)	5.40	6.22	6.55
*ρ*_DC_ at RT (Ωm)	5.2 × 10^6^	1.1 × 10^8^	5.4 × 10^7^
*ε* at RT	1265	1125	785
tan*δ* at RT	0.1	0.034	0.013
*E*_a_ (eV) at high *T*	1.17	1.13	1.00
*E*_a_ (eV) at low *T*	1.27	0.48	0.84
*P*_r_ at RT (µC/cm^2^)	5.40	3.13	2.36
*P*_s_ at RT (µC/cm^2^)	7.90	5.54	4.22
*E*_c_ at RT (kV/mm)	0.68	0.76	0.76

RT—test at room temperature.

## Data Availability

Data are contained within the article.

## Supplementary Materials

The following supporting information can be downloaded at https://www.mdpi.com/article/10.3390/ma17010049/s1, Figures S1–S3: X-ray diffraction patterns BaTiO_3_ material, Pb_0.94_Sr_0.06_ (Zr_0.46_Ti_0.54_)_0.99_Cr_0.01_O_3_ material, and Ni_0.64_Zn_0.36_Fe_2_O_4_ ferrite, respectively.
