# Supplementary material for: Electrophysical Properties of the Three-Component Multiferroic Ceramic Composites
Source: Materials (Basel). 2023 Dec 22;17(1):49. doi: 10.3390/ma17010049 (PMC10779473; doi:10.3390/ma17010049)
Supplement: Supplementary file 1 [file materials-17-00049-s001.zip › materials-2759957-supplementary.pdf]

Supplementary materials

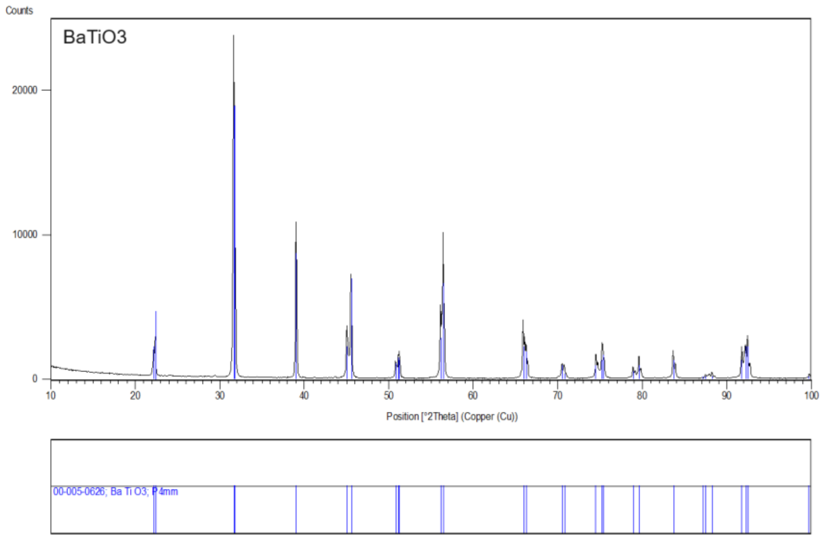

Figure S1. X-ray diffraction patterns for $BaTiO_3$ material.

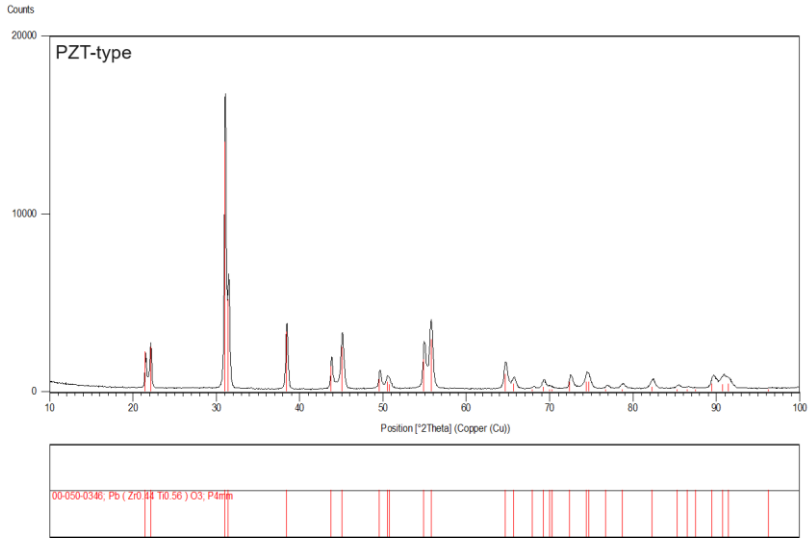

Figure S2. X-ray diffraction patterns for $Pb_{0.94}Sr_{0.06}(Zr_{0.46}Ti_{0.54})_{0.99}Cr_{0.01}O_3$ material.

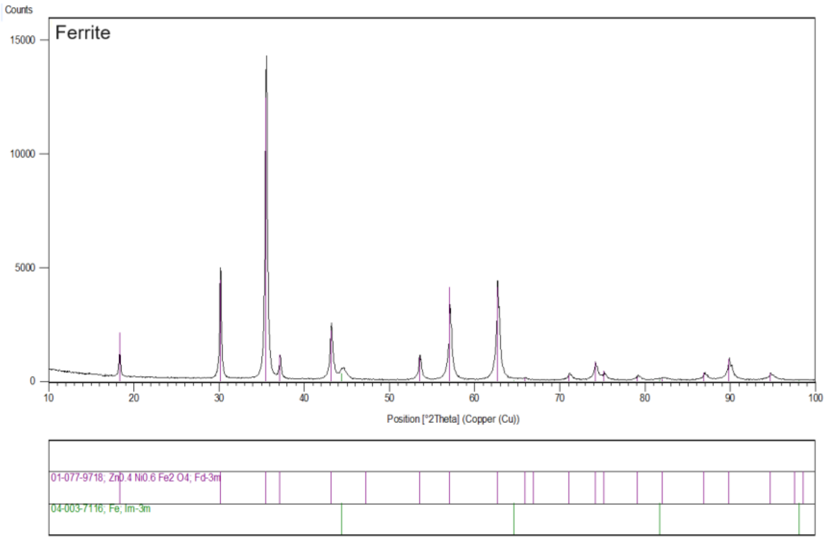

Figure S3. X-ray diffraction patterns for $Ni_{0.64}Zn_{0.36}Fe_2O_4$ ferrite material.
